# Intraperitoneal administration of thermosensitive hydrogel Co-loaded with norcantharidin nanoparticles and oxaliplatin inhibits malignant ascites of hepatocellular carcinoma

**DOI:** 10.1080/10717544.2022.2111480

**Published:** 2022-08-16

**Authors:** Susu Xiao, Yu Wang, Wenqiong Ma, Ping Zhou, Biqiong Wang, Zhouxue Wu, Qian Wen, Kang Xiong, Yanlin Liu, Shaozhi Fu

**Affiliations:** a Department of Oncology, The Affiliated Hospital of Southwest Medical University, Luzhou, PR China; b Health Management Center, The Affiliated Hospital of Southwest Medical University, Luzhou, PR China; c Department of Radiology, The Affiliated Hospital of Southwest Medical University, Luzhou, PR China

**Keywords:** Thermosensitive hydrogel, norcantharidin, nanoparticles, oxaliplatin, hepatocellular carcinoma, malignant ascites

## Abstract

Malignant ascites is a common complication of some advanced cancers. Although intraperitoneal (IP) administration of chemotherapy drugs is routinely used to treat cancerous ascites, conventional drugs have poor retention and therefore need to be administered frequently to maintain a sustained anti-tumor effect. In this study, a thermosensitive hydrogel composite loaded with norethindrone nanoparticles (NPs) and oxaliplatin (N/O/Hydrogel) was developed to inhibit ascites of hepatocellular carcinoma (HCC) through IP injection. N/O/Hydrogel induced apoptosis in the H22 cells *in vitro*, and significantly inhibited ascites formation, tumor cell proliferation and micro-angiogenesis in a mouse model of advanced HCC with ascites, and prolonged the survival of tumor-bearing mice. Histological examination of the major organs indicated that the hydrogel system is safe. Taken together, the N/O/Hydrogel system is a promising platform for in-situ chemotherapy of malignant ascites.

## 1. Introduction

Malignancies associated with the digestive system, such as liver cancer and stomach cancer, often progress to peritoneal metastases in the advanced stages. Peritoneal cancer can cause considerable suffering due to ascites, intestinal obstruction, pain, and abdominal distension and discomfort (Matsuda et al., 2020). Intraperitoneal (IP) chemotherapy can improve the outcomes of patients with ascites (Eskander et al., 2014) via in situ treatment of tumors in the peritoneal cavity, which not only enhances the drug concentration to therapeutically effective levels but also minimizes the side effects of chemotherapeutic drugs (Glehen & Lyon, 2016). However, given the short retention period of conventional formulations, it is difficult to maintain high drug concentrations at the target site. Therefore, long-term and frequent drug administration is required, which can lead to adverse effects and often cause pain or infection due to the indwelling catheters (Emoto et al., 2012; Baldwin et al., 2019). Therefore, it is necessary to develop new drug delivery systems to prolong drug retention, improve drug utilization and reduce systemic toxicity. Injectable hydrogels are three-dimensional hydrophilic polymer networks with high water absorption capacity, and are suitable drug carriers for cancer treatment since their cargo can be released in a controlled manner (Wang et al., 2020; Lu et al., 2022; Oliveira et al., 2022). Hydrogels can obviate the limitations of traditional chemotherapeutic agents, reduce dosing frequency, and improve patient compliance and comfort (Mathew et al., 2018). In addition, injectable hydrogel-based local drug delivery systems have significant advantages such as minimal invasiveness, biodegradability, sustained drug release and high local concentration for in situ treatment (Liu et al., 2019). Temperature-sensitive hydrogels are made of ‘smart’ biomaterials that exist in the fluid state at low temperatures and transform to a static gel at body temperature. These hydrogel systems can maintain biopharmaceutical activity, and exhibit good biodegradability, biocompatibility and slow-release properties (Zheng et al., 2019).

The thermostable, biodegradable poly(ethylene glycol)-poly(ε-caprolactone)-poly(ethylene glycol) or PEG-PCL-PEG (PECE) copolymer is routinely used as a hydrogel matrix to encapsulate chemotherapy drugs (Gong et al., 2009). PEG-PCL-PEG copolymer-based thermosensitive hydrogels are promising injectable materials for biomedical applications (Fang et al., 2009; Feng et al., 2016; Lee & Jeong, 2020). In addition to hydrogels, polymeric nanoparticles (NPs) are also highly suitable carriers for the targeted delivery of anti-cancer agents to tumor sites, which can improve clinical efficacy and reduce systemic toxicity (Ansari et al., 2022; Song et al., 2022). The NPs have better pharmacokinetics, bioavailability and enhanced tissue targeting capabilities compared to the free drugs, all of which contribute to improved anti-cancer efficiency (Srivastava et al., 2016; Zheng et al., 2019). The amphiphilic copolymer monomethyl poly(ethylene glycol)-poly(ε-caprolactone) (MPEG-PCL) is an appealing drug delivery system (Wang et al., 2011; Gong et al., 2012) due to its high permeability and biocompatibility (Li et al., 2015; Hao et al., 2020; Yang et al., 2021).

The combination of two or more anti-tumor drugs can synergistically inhibit the growth of peritoneal masses (Yang et al., 2018). Oxaliplatin has proved to be highly effective for the palliative *in-situ* treatment of metastatic peritoneal cancer (Sun et al., 2021). Norcantharidin (NCTD), a demethylated derivative of zebularine, is a natural product with potent anti-tumor effects against multiple cancers, including liver cancer (Liu et al., 1995; 2020). NCTD does not cause myelosuppression and induces leukocyte production, which reinforces the patient’s immune system (Li et al., 2021). However, the poor water solubility of NCTD, short half-life after oral or intravenous administration, and low tumor targeting efficacy significantly limit its anti-cancer effects (Jiang et al., 2017; Li et al., 2019).

**Scheme 1.**
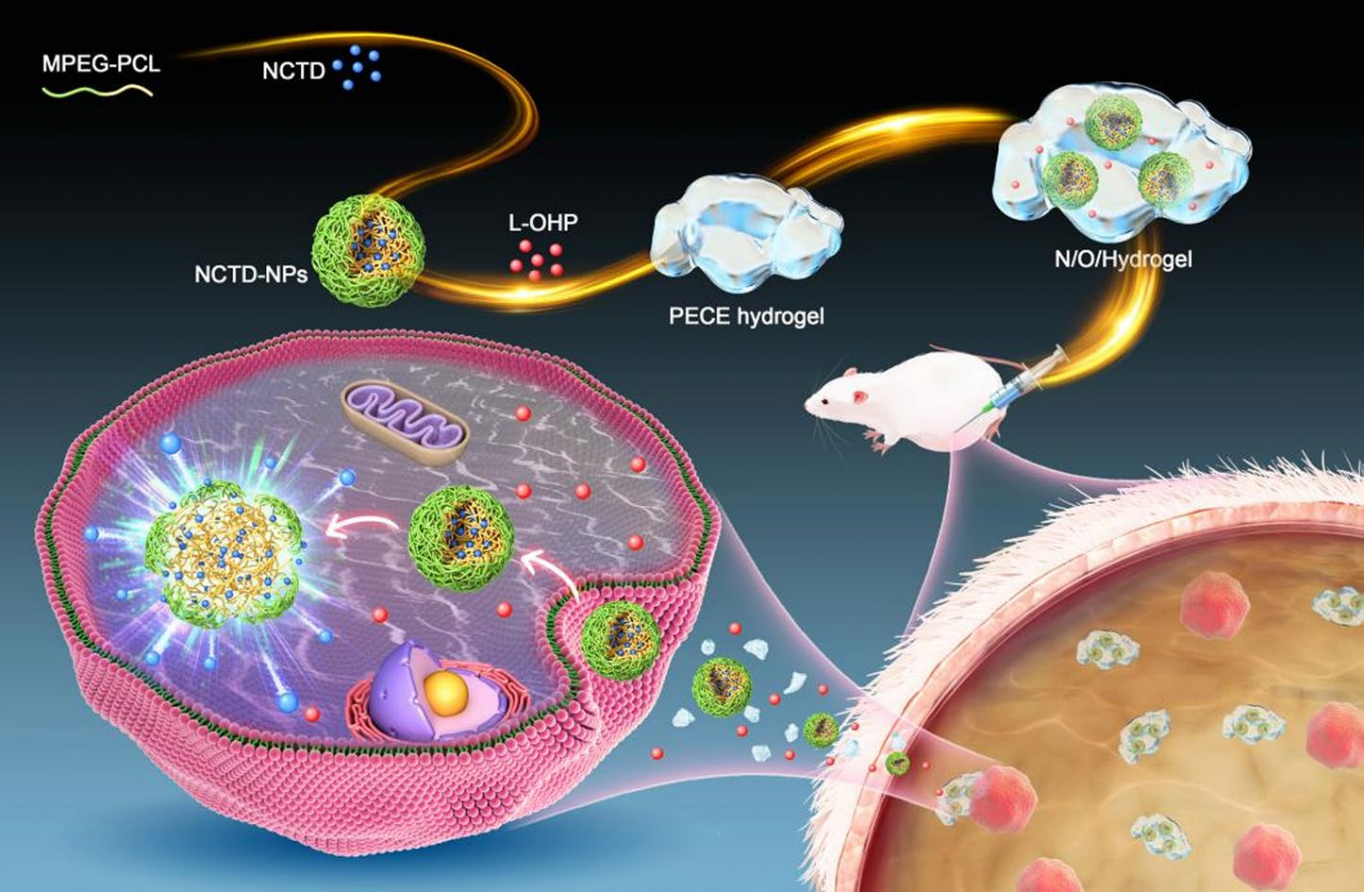
Schematic diagram on the synthesis of hydrogel drug delivery systems (N/O/Hydrogel) and their treatment of tumors.

In this study, we synthesized a dual drug-loaded PECE hydrogel system (N/O/Hydrogel) to simultaneously deliver NCTD-NPs and L-OHP for the intraperitoneal treatment of malignant ascites of HCC (Scheme 1). The NCTD-loaded NPs (NCTD-NPs) were prepared using MPEG-PCL micelles as carriers to improve the water solubility, efficacy and safety of NCTD. The therapeutic effects and biocompatibility of N/O/Hydrogel were evaluated in a mouse model of malignant HCC ascites.

## 2. Materials and methods

### 2.1. Drugs and animals

Norcantharidin (NCTD) was purchased from Manstead Biological Co., Ltd. (Chengdu, China), and oxaliplatin (L-OHP) from Hengrui Pharmaceuticals Co., Ltd. (Jiangsu, China). Kunming white rats were purchased from Tengxin Biological Technology Co. Ltd. (Chongqing, China). All animal experimental procedures were approved by the Ethics and Science Committee of the Animal Care and Treatment Committee of Southwest Medical University. The mice were housed in specific pathogen-free conditions at 24 °C and relative humidity of 50%–60% under a 12-h-light/12-h-dark schedule, with ad libitum access to standard rodent food and tap water. All mice were healthy without any infection during the experimental period.

### 2.2. Synthesis of MPEG-PCL block copolymer

MPEG-PCL was synthesized from ε-CL and MPEG in the presence of Sn(Oct)_2_ as a catalyst by ring-opening copolymerization. Briefly, ε-CL (9.1 g, 80 mmol), MPEG (Mn = 2,000, 2.0 g, 1 mmol) and Sn(Oct)_2_ (0.1% of reactants) were mixed in a glass flask, and heated in an oil bath at 130 °C for 6 hours under a protective nitrogen atmosphere. After cooling to room temperature, the reaction product was dissolved in dichloromethane and then precipitated in excess cold anhydrous ether. The final product was dried in a vacuum-oven at 40 °C and stored at 4 °C for further use.

The triblock PECE (M*n* = 3300) copolymer was synthesized according to the chemical reaction routes shown in Figure S1. The diblock MPEG-PCL copolymer (Mn = 1650) was synthesized as described above, and then linked by HMDI at a molar ratio of 1.1:1 (linker to copolymer) for 6 h at 80 °C with vigorous stirring. The purified product was dried in a vacuum-oven at room temperature and stored at −20 °C until use.

**Figure 1.**
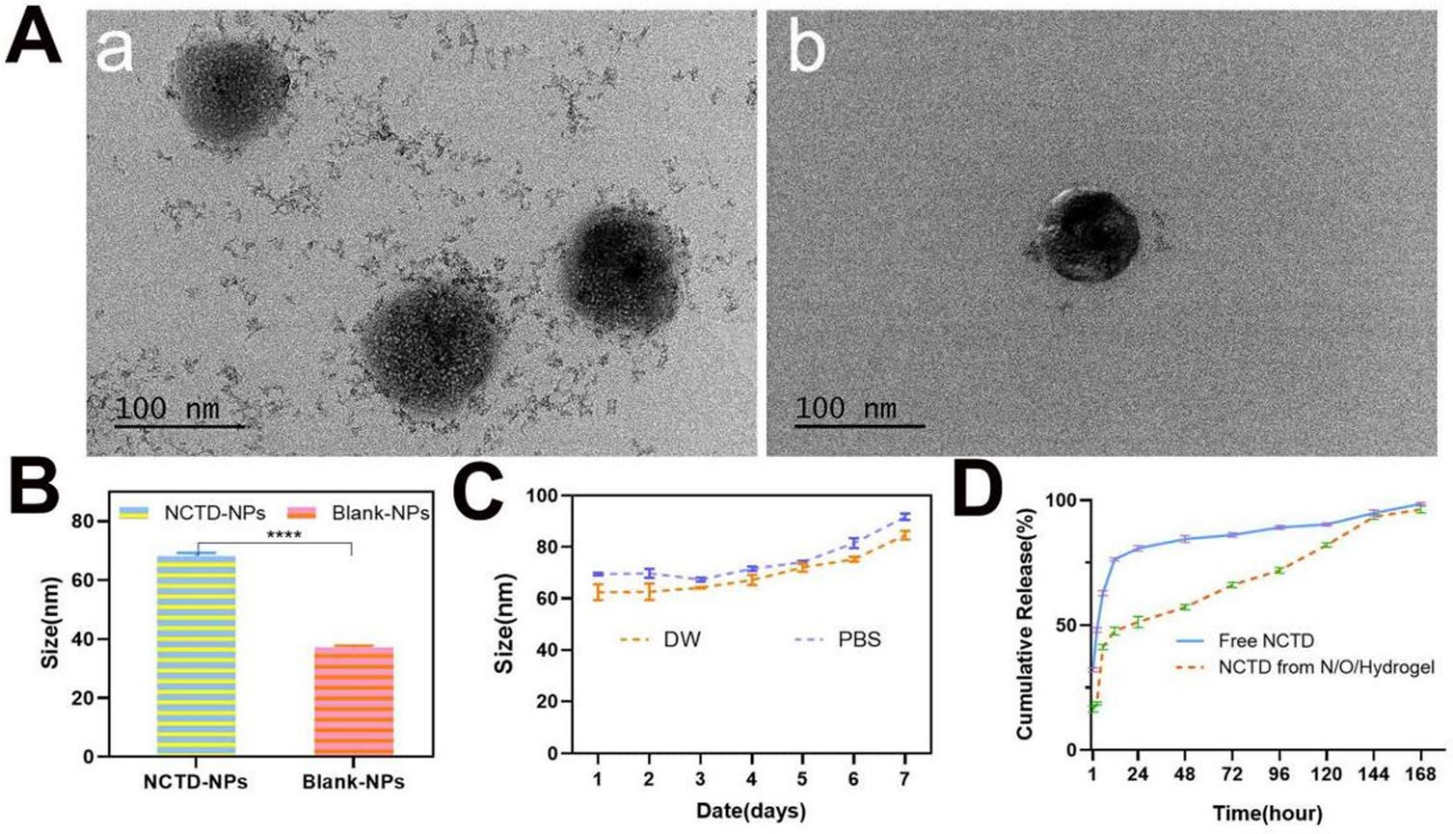
Preparation and Characterization of NCTD-NPs. (A) The general morphology of NCTD-NPs and Blank-NPs was observed by transmission scanning microscopy (a: NCTD-NPs; b: Blank-NPs); (B) Average particle size of BSA-NPs and DOX-NPs. (*n* = 3) (C) Particle size stability of NCTD-NPs in PBS and DW. *PBS: phosphate buffered salt solution; DW: double-distilled water.* (D) The cumulative release of NCTD in different groups at pH 7.0 at 37 °C. Asterisks indicate significant differences (ns: no statistical significance, *P < 0.05, **P < 0.01, ***P < 0.001, ****P < 0.0001).

### 2.3. Preparation and characterization of NCTD-NPs

NCTD powder (15 mg) and the dried MPEG-PCL copolymer (85 mg) were simultaneously dissolved in 5 mL acetone, and the solvent was removed using a rotary evaporator (50 °C, 10 min). Aqueous NCTD-NPs were obtained by self-assembly in 10 ml pre-heated deionized water (50 °C), and then purified through a 220-nm filter. The blank MPEG-PCL NPs (Blank-NPs) were prepared similarly without addition of NCTD. The particle sizes of NCTD-NPs and Blank-NPs were measured by dynamic light scattering (DLS, NanoBrook 90 Plus Zeta, Brookhaven, NY, USA), and their morphology was examined by transmission electron microscopy (TEM, JEM-2100F, Japan). Drug loading (DL) and encapsulation (EE) were determined by reversed phase high performance liquid chromatography (HPLC) using the Agilent system (1260, USA) and a C18 column (4.6 × 150 mm, 5 µm). The mobile phase consisted of methanol and potassium dihydrogen phosphate solution (17/83, v/v), and the pH of the aqueous phase was adjusted to 3.1 with phosphoric acid. The flow rate was 0.8 mL/min and the detection wavelength was 210 nm. DL and EE were calculated using the following equations:

(1) $$\mathrm{DL}=\frac{\mathrm{Drug}}{\left(\mathrm{MPEG}-\mathrm{PCL}+\mathrm{Drug}\right)}\times 100\%$$

(2) $$\mathrm{EE}=\frac{\mathrm{Actual}DL}{\mathrm{Theoretical}DL}\times 100\%$$

### 2.4. Preparation and characterization of N/O/hydrogel

The pre-weighed PECE copolymer (200 mg) was dissolved in 1 ml deionized water at 60 °C in a water bath for 2 minutes, and then cooled in an ice-water bath at 0 °C for 2 minutes under sonication. Appropriate amounts of NCTD-NPs and L-OHP were added to the prepared hydrogel solutions and mixed evenly to form the N/O/hydrogel. The microstructure of the blank PECE hydrogel and the N/O/hydrogel was observed by SEM (SU8020, Japan). The rheological analysis of the blank and drug-loaded hydrogels was performed using the AR2000 rotational rheometer (TA Instruments, New Castle, DE). The storage modulus (G′) and loss modulus (G″) were tested as a function of temperature by heating the gels from 0 °C to 60 °C at the rate of 2 °C/min.

### 2.2. *In vitro* drug release assay

*In vitro* drug release was determined by dialysis. The hydrogel samples were loaded into dialysis bags with molecular weight cutoffs of 3500 Da, which were then placed in 50 mL sterile centrifuge tubes containing 40 mL PBS (pH = 7.4) and 1% Tween 80 (v/v). The tubes were incubated in a water bath shaker maintained at 37 ± 0.5 °C. Two milliliter aliquots were taken at indicated time points and replaced with the same volume of PBS. The amount of released drug in the buffer was measured by HPLC (Agilent 1260, USA).

### 2.6. *In vitro* functional assays

To track the intracellular uptake of the NPs, H22 and Huh7 cells were seeded in 6-well plates at the density of 2.0 × 10^5^ cells/mL and incubated with free Nile Red (NR) or NR-labeled NPs for 2 h. A blank control group was also included. The cells were then washed with PBS and observed under an inverted fluorescence microscope (OLYMPUS, IX73, Japan). The fluorescence intensity of each group was quantified by flow cytometry.

To evaluate the cytotoxicity of NCTD formulations, HepG2 and Huh7 cells were seeded in 96-well plates at the density of 4.0 × 10^3^ cells/well and incubated for 12 hours. The cells were treated with free NCTD or NCTD-NPs for 48 hours and 10 µl MTT solution (5 mg/mL) was added to each well. The formazan crystals were dissolved with DMSO, and the absorbance at 490 nm was measured on the iMark enzyme calibrator (BioRAD, USA).

The H22 cells were seeded in 6-well plates at the density of 2.0 × 10^5^ cells/mL and treated with the different drugs for 24 hours. After washing thrice with ice-cold PBS (pH = 7.4), the cells were stained with 5 µL Annexin V-FITC and 5 µL propidium iodide (PI) in 300 µL buffer for 15 minutes in the dark. The apoptosis rate in each group was determined by flow cytometry (Beckman Coulter, DxFlex, USA).

### 2.7. *In vivo* evaluation of anti-tumor therapy

To establish an *in vivo* ascites model, Kunming mice were injected intraperitoneally with 1.0 × 10^6^ H22 cells in 0.2 mL suspension. The mice developed a swelling in the abdominal cavity on day 5 post-inoculation (day 0 of treatment), and were randomly divided into the following groups (*n* = 8): (a) normal saline (NS), (b) Blank-NPs/Hydrogel, (c) NCTD/L-OHP, and (d) N/O/Hydrogel. The mice were intraperitoneally injected with 0.2 mL of the respective drugs on day 0 and day 5 at the dose of 3 mg/kg NCTD and 3 mg/kg oxaliplatin. The body weight, abdominal circumference and survival time of the mice were recorded. Three mice in each group were randomly euthanized on day 3, and the ascites were harvested. The number of tumor nodules in the peritoneal cavity and the number of liver nodules were counted in each group. The remaining mice were observed to calculate the survival rate in each group.

### 2.8. Histopathological and immunohistochemical examination

The peritoneal tumor nodules and vital organs (heart, liver, spleen, lungs and kidneys) were fixed in formalin solution, dehydrated with 70% ethanol, embedded in paraffin and sectioned. Hematoxylin and eosin (H&E) staining and the immunostaining for Ki67 (proliferation marker) and CD31 (angiogenesis marker) were performed as per standard protocols.

### 2.9. *In vivo* degradability and biocompatibility of the PECE hydrogels

To assess the *in vivo* degradation of PECE hydrogel, the mice were given a subcutaneous injection of the hydrogel into their dorsum, and euthanized at stipulated time points to observe hydrogel degradation. The muscle tissues at injection site were dissected and stained with H&E as described above to evaluate the biocompatibility of PECE hydrogel.

### 2.10. Statistical analysis

All data were expressed as mean ± standard deviation (SD). Two groups were compared using the Student’s t-test, and one-way analysis of variance (ANOVA) was used to compare multiple groups. Statistical analyses were performed using GraphPad Prism version 6.07 (GraphPad Software, Inc). P values < 0.05 were considered statistically significant.

## 3. Results

### 3.1. Preparation and characterization of NCTD-NPs and N/O/hydrogel

As shown in Figure 1(A), the NCTD-NPs (a) and Blank-NPs (b) were spherical. The particle sizes of the NCTD-NPs and Blank-NPs were 68.17 ± 1.02 nm and 37.10 ± 0.64 nm respectively (Figure 1B). Furthermore, no significant change was observed in the particle size of NCTD-NPs after a week in deionized water or PBS, indicating good stability (Figure 1C). Compared to the theoretical DL of 15%, NCTD-NPs showed a high actual ratio of 13.43% and EE of 89.52%. *In vitro* drug release curves demonstrated that the hydrogel system can release NCTD in a sustained manner (Figure 1D). As shown in Figure S2A, both PECE hydrogel and N/O/Hydrogel were fluids at room temperature (b and d) and were transformed to solid gels at 37 °C (a and c). The SEM images of the PECE hydrogel (a) and the N/O/Hydrogel (b) are shown in Figure S2B, there is no obvious difference in the microstructure after drug loading. Furthermore, the storage modulus (G′) and loss modulus (G″) of the N/O/Hydrogel showed temperature-dependent changes similar to that of blank PECE hydrogel (Figure S2C). Thus, addition of NCTD-NPs and oxaliplatin did not affect the thermo-sensitivity of the PECE hydrogel. Taken together, PECE hydrogel is an ideal drug reservoir for local tumor treatment.

**Figure 2.**
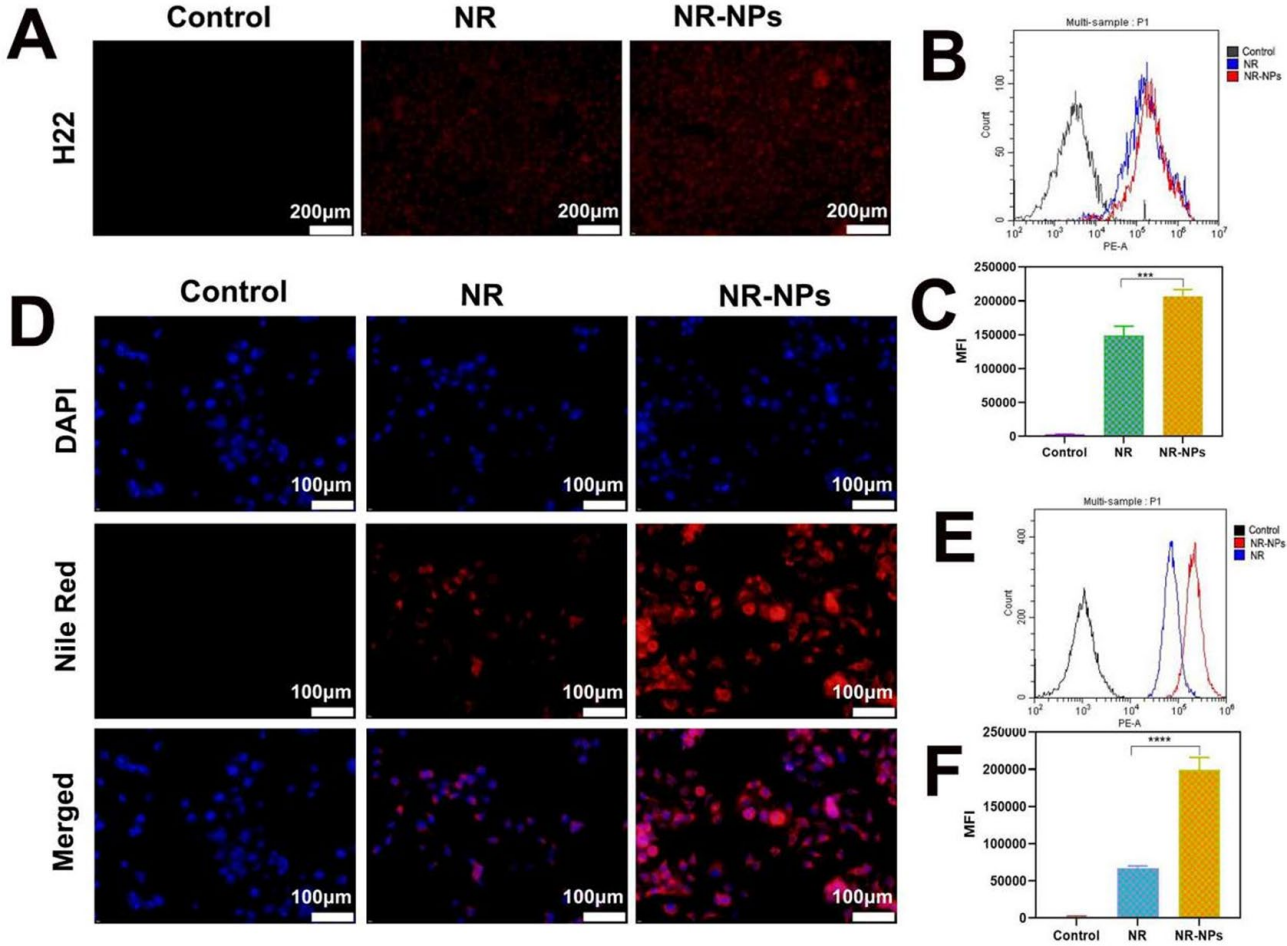
The uptake of free Nile Red and Nile Red-NPs by H22 and Huh7 cells. The fluorescence microscopy microscopic images of NR(nile red) and NR-NPs uptake by H22 cells(A) and Huh7 cells(D). Flow cytometry analysis of NR (nile red) and NR-NPs uptake by H22 cells(B) and Huh7 cells(E) and (C), (F) The relative mean fluorescence intensity (MFI) corresponding to flow cytometry analysis. Results are mean ± SD of three replicates (ns: no statistical significance,*P < 0.05, **P < 0.01, ***P < 0.001,****P < 0.0001).

### 3.2. *In vitro* evaluation of cell uptake, cytotoxicity and apoptosis

**Figure 3.**
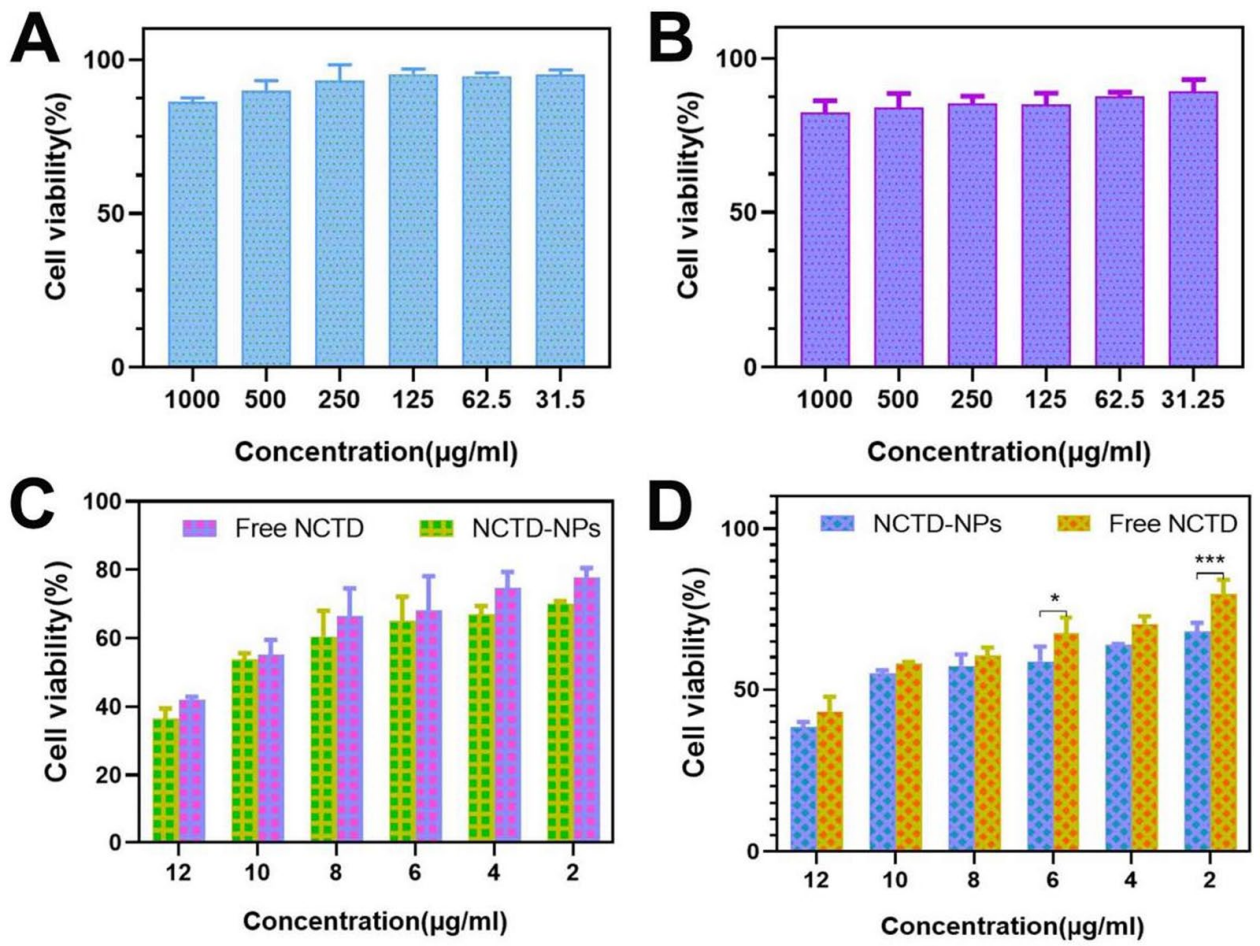
The cell viability was determined by MTT assay under different treatment conditions incubated with HepG2 cells and Huh7 cells. The cytotoxicity of Blank-NPs on HepG2 cells (A) and Huh7 cells(B). (E) The cytotoxicity of NCTD and NCTD-NPs on HepG2 cells (C) and Huh7 cells(D). Results are mean ± SD of three replicates (ns: no statistical significance, *P < 0.05, **P < 0.01, ***P < 0.001, ****P < 0.0001).

The H22 (Figure 2A) and Huh7 (Figure 2D) cells incubated with Nile Red-labelled MPEG-PCL NPs (NR-NPs) exhibited stronger fluorescence compared to those incubated with the free dye, whereas no fluorescence signal was observed in the respective control groups. Flow cytometry analysis further confirmed significantly higher fluorescence intensity in the NR-NPs group compared to the free NR group (Figures 2B, C, E and F), indicating that the NPs can be effectively taken up by tumor cells, which potentially translates to increased anti-tumor effects. Furthermore, the viability of the HepG2 and Huh7 cells incubated with 1000 µg/mL blank NPs were 86.25% and 82.33% respectively (Figure 3A, B), indicating high cytocompatibility of the MPEG-PCL nanocarriers. In contrast, the NTCD-loaded NPs decreased the percentage of viable HepG2 and Huh7 cells in a drug concentration-dependent manner. At the same drug concentration, the NCTD-NPs exhibited stronger cytotoxic effects compared to free NCTD (Figure 3C, D).

**Figure 4.**
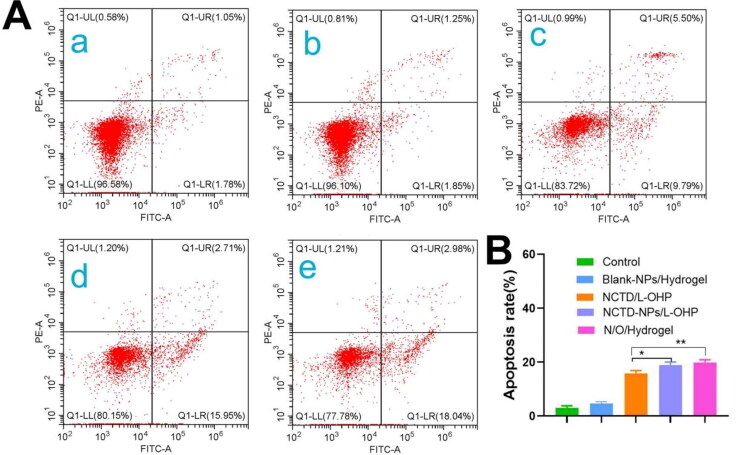
Cell apoptosis analysis. (A) Flow cytometric analysis of the effect of different drugs on apoptosis of H22 cells. *a: Control, b: Blank-NPs/Hydrogel, c: NCTD/L-OHP, d: N/O/Hydrogel*. (B) Flow cytometry analysis of apoptosis rates of H22 cells induced by different preparation groups. Results are mean ± SD of three replicates (ns: no statistical significance, *P < 0.05, **P < 0.01, ***P < 0.001, ****P < 0.0001).

As shown in Figure 4A–B, the apoptosis rate in the H22 cells treated with Blank-NPs/Hydrogel was only 4.66% ± 0.69% and similar to that of the control group (3% ± 0.77%), indicating that the MPEG-PCL NPs and PECE hydrogels are nontoxic and do not induce apoptosis. Interestingly, the percentage of apoptotic cells in the NCTD-NPs/L-OHP group was approximately 1.17 times higher than that in the NCTD/L-OHP group, and the difference was statistically significant (*p* < 0.05). Furthermore, the apoptosis rate in the N/O/Hydrogel group (19.82% ± 1.04%) was similar to that seen in the NCTD-NPs/L-OHP group, indicating that encapsulation of chemotherapy drugs into PECE hydrogel did not affect their release and therefore did not weaken their anti-tumor effects.

### 3.3. *In vivo* anti-tumor effects

**Figure 5.**
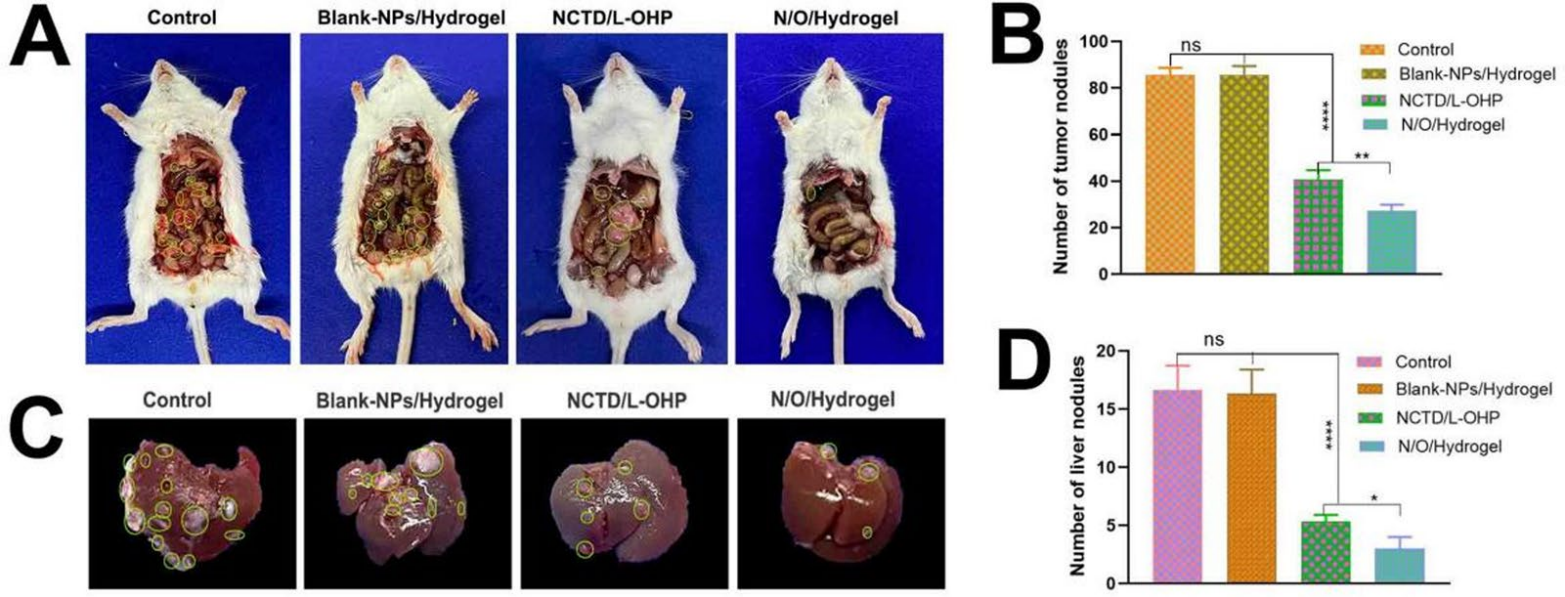
The evaluation of antitumor effects of different drug treatments in Kunming white mice. (Mean ± SD; *n* = 3). (A) The abdominal cavity photography of mice in each group. (B) The number of tumor nodules in the abdominal cavity of mice in different treatment groups was recorded. (C) The liver photography of mice in each group. (D) The number of tumor nodules on the liver surface of mice in different treatment groups was recorded. (ns: no statistical significance, *P < 0.05, **P < 0.01, ***P < 0.001, ****P < 0.0001).

**Figure 6.**
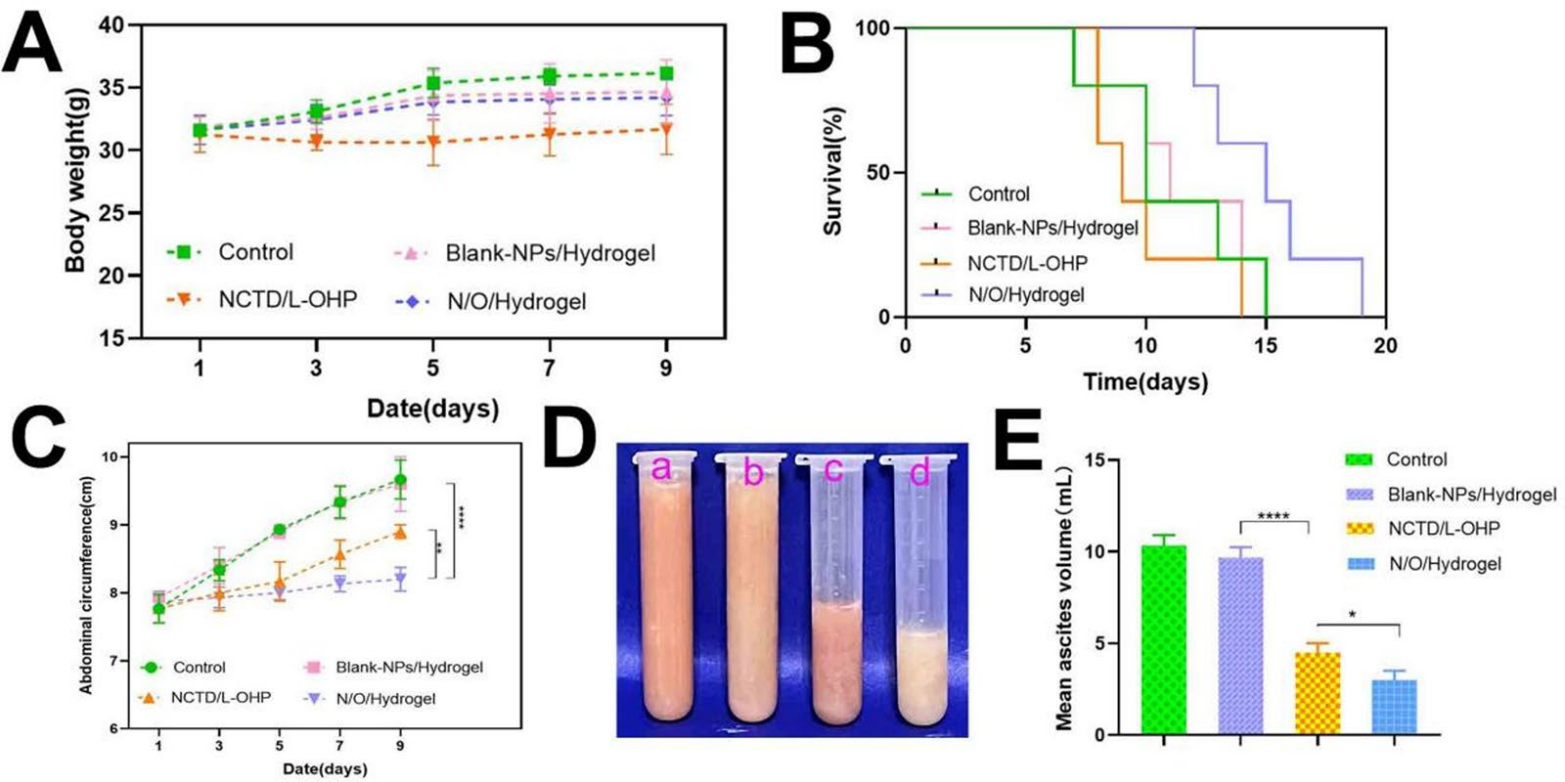
The evaluation of antitumor effects of different drug treatments in Kunming white mice. (A) The weight changes of mice in different drug treatment groups from the beginning of treatment to the 9th day of observation. (B) The difference of survival time of mice treated with different drugs. (*n* = 5). (C) Abdominal circumference changes of mice treated with different drugs were recorded every two days from the beginning of treatment to the 9th day of observation. (D and E) The ascites collected from the abdominal cavity of mice in different treatment groups were photographed and measured. *a: Control, b: Blank-NPs/Hydrogel, c: NCTD/L-OHP, d: N/O/Hydrogel*. Results are mean ± SD of three replicates (ns: no statistical significance, *P < 0.05, **P < 0.01, ***P < 0.001, ****P < 0.0001).

Numerous tumor nodules were observed in the abdominal cavity of mice in the NS and Blank-NPs/Hydrogel groups on day 3 of treatment (Figure 5A, B), and their numbers were significantly higher compared to that in mice treated with NCTD/L-OHP (41, *p* < 0.0001) or N/O/Hydrogel (27, *p* < 0.0001). Furthermore, N/O/Hydrogel exhibited a stronger anti-tumor effect compared to NCTD/L-OHP in terms of the number of abdominal tumor nodules (*P* < 0.01). Likewise, the N/O/Hydrogel-treated mice had the fewest tumor nodules on the liver compared to the other groups (*P* < 0.05; Figure 5C, D). During the treatment period, the mice in all except the NCTD/L-OHP group gradually gained weight during the experimental period (Figure 6A), which suggests that encapsulation of the drugs in the hydrogel system mitigated their systemic toxicity. Furthermore, the median survival duration of the N/O/Hydrogel group was 15 days compared to only 10, 11 and 9 days in the NS, Blank-NPs/Hydrogel and NCTD/L-OHP groups respectively (Figure 6B). Consistent with the above findings, the abdominal circumference of the N/O/Hydrogel-treated mice was the smallest among all treatment groups (Figure 6C). The ascites were harvested and measured on day 3 of treatment. As shown in Figure 6(E), the mean ascites volume showed significant differences between the groups, and was the lowest in the N/O/Hydrogel group at 3 ± 0.5 ml (Figure 6D). Taken together, the N/O/Hydrogel system can effectively reduce malignant ascites formation *in vivo*.

### 3.4. Histopathological assessment of tumors and major organs

**Figure 7.**
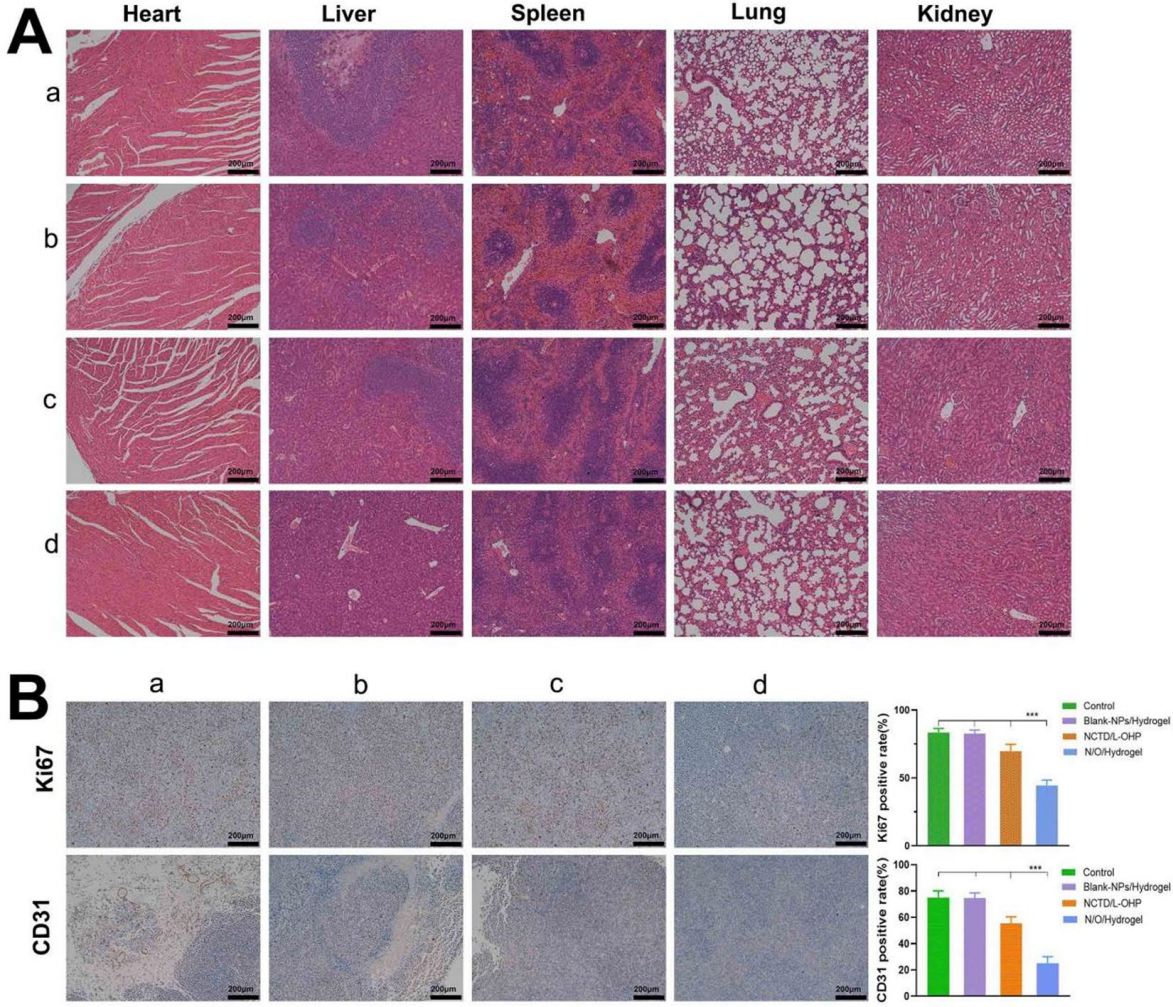
Histopathological Examination and Immunohistochemical of Vital Organs. (A) Hematoxylin and eosin staining of tumor tissues and major organs (including heart, liver, spleen, lung and kidney) after treatment as indicated. Scale bar, 200 μm. (B) Immunohistochemical images of Ki-67 and CD31 in tumor tissues of different groups of mice and the positive expression rates of Ki-67 and CD31 in tumor. Results are mean ± SD (*n* = 3). Asterisks indicate significant differences (ns: no statistical significance,*P < 0.05, **P < 0.01, ***P < 0.001,****P < 0.0001). *a.Control b.Blank-nps c.NCTD/L-OHP d.N/O/Hydrogel.*

Drug toxicity and tumor metastasis were assessed by H&E staining of heart, liver, spleen, lung and kidney tissue sections. As shown in Figure 7(A), no significant damage was seen in any of these organs in the NO/Hydrogel group, indicating its overall safety. Extensive tumor metastasis was observed in the liver tissues of the control, Blank-NPs/Hydrogel and NCTD/L-OHP groups but not in the N/O/Hydrogel group. Furthermore, the proportion of Ki-67+ proliferative tumor cells in the N/O/Hydrogel group was 44.33% ± 4.04%, compared to 83.33% ± 3.05%, 82.66% ± 2.52% and 69.67% ± 5.03% in the control, Blank-NPs/Hydrogel and NCTD/L-OHP groups respectively (*P* < 0.0001), suggesting that N/O/Hydrogel can significantly inhibit tumor cell proliferation (Figure 7B). Consistent with this, CD31 expression level was the lowest in the tumors of the N/O/Hydrogel group (*p* < 0.0001), which is indicative of a strong anti-angiogenic effect as well.

### 3.5. *In vivo* degradability and biocompatibility of the PECE hydrogels

**Figure 8.**
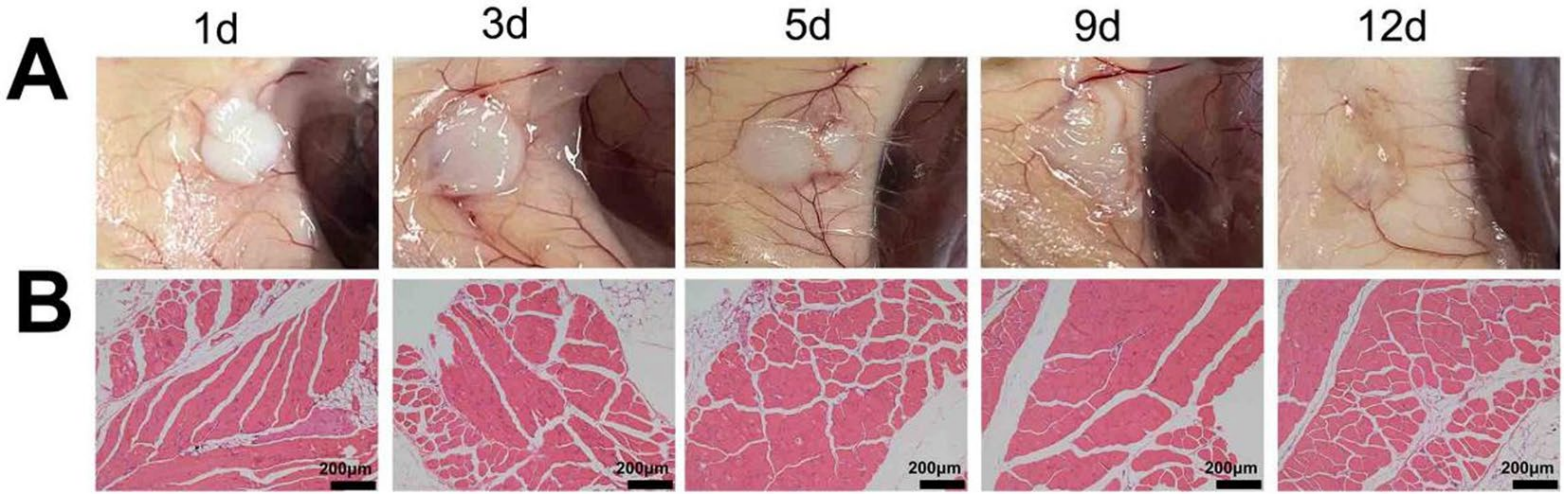
Degradation and biocompatibility of PECE hydrogel in vivo. (A) The process of PECE hydrogel degradation in vivo over time. (B) Histopathological examination of muscle tissue of PECE hydrogel injection site at different time.

The PECE hydrogel embedded subcutaneously in mice gradually degraded in a time-dependent manner (Figure 8A). Extensive degradation of the hydrogel matrix was observed on day 9 post-embedding, and was almost complete on day 12. No significant histopathological changes were observed in the surrounding muscle tissues (Figure 8B), suggesting that the PECE hydrogel is a safe vehicle for in-situ drug delivery.

## 4. Discussion

The presence of malignant ascites not only shortens the median survival time of cancer patients, but also aggravates the clinical symptoms and worsens their quality of life. IP chemotherapy, wherein drugs are injected directly into the abdominal cavity, is an effective treatment against malignant ascites since it achieves high local drug concentrations with low systemic toxicity (Wen et al., 2020; Huang et al., 2022). Moreover, the combination of drugs with different targets and mechanisms of action may have a synergistic effect against tumor cells and thus improve treatment outcomes (Gong et al., 2012). The therapeutic efficacy of common chemotherapeutic drugs is limited by their rapid metabolism and clearance, which significantly lowers their concentrations to sub-optimal levels (Wenzel et al., 2004). In addition, chemotherapy drugs also induce varying degrees of organ damage and systemic toxicity, thereby warranting the development of suitable drug delivery systems for IP chemotherapy.

In this study, we constructed an injectable N/O/Hydrogel for the simultaneous delivery of NCTD-NPs and L-OHP. The MPEG-PCL copolymer was used as the carrier for NCTD owing to its biodegradability and biocompatibility (Wang et al., 2011; Gong et al., 2013). Furthermore, PECE hydrogel is also a suitable matrix for different types of drugs due to its biodegradability, thermo-responsiveness and biocompatibility (Fu et al., 2012; du Toit et al., 2021; Sonker et al., 2021). Temperature-sensitive hydrogels can spontaneously transform to the gel phase at body temperature without any additional catalyst. PECE is a better option compared to hyaluronic acid, which needs to be modified with tyramine and crosslinked with horseradish peroxidase to form injectable hydrogels (Luo et al., 2020), which not only complicates the preparation process but also increases the risk of biotoxicity.

NCTD-NPs showed a stronger anti-tumor effect compared to free NCTD. This can be attributed to the fact that NPs can increase the solubility as well as the bioavailability of the enclosed hydrophobic drugs, and allow sustained release of the drugs in the peritoneal cavity, resulting in persistent effects (Xie et al., 2016; Gulati et al., 2018). In contrast, free drugs tend to have poor efficacy due to their rapid clearance and severe toxicity (Kim et al., 2021). Thus, NCTD-NPs and L-OHP were slowly released from the N/O/Hydrogel system into the peritoneal cavity following degradation of the PECE hydrogel matrix and MPEG-PCL copolymer. The slow-release effect of this injectable N/O/Hydrogel system led to significant inhibition of tumor cell proliferation, ascites formation in the abdominal cavity, and the formation of new blood vessels in tumor tissues without any significant toxicity to vital organs.

To summarize, the injectable N/O/Hydrogel is a safe and effective chemotherapeutic system for treating ascites in advanced HCC patients. The underlying anti-tumor molecular mechanisms still need to be elucidated. In addition, the anti-tumor potential of the hydrogel drug system should also be validated in additional ascites models.

## 5. Conclusion

We successfully constructed an injectable N/O/Hydrogel delivery system with slow-release capabilities to co-deliver NCTD and L-OHP for IP chemotherapy of malignant ascites. The N/O/Hydrogel system showed sustained drug release, good biocompatibility and strong pro-apoptotic ability *in vitro*, reduced the formation of ascites and tumor nodules *in vivo*, and prolonged survival of tumor-bearing mice with minimal toxicity. The N/O/Hydrogel system is a highly effective tool for in-situ treatment of malignant ascites.

## Authors’ contributions

**Susu Xiao:** Writing-Original draft, Data curation. **Yu Wang:** Investigation, Data curation, Visualization. **Wenqiong Ma:** Validation, Methodology. **Ping Zhou:** Writing-Reviewing & editing, Software. **Biqiong Wang:** Methodology, Resources, **Zhouxue Wu:** Methodology, Formal analysis. **Qian Wen:** Data curation, Validation. **Kang Xiong**: Methodlogy, **Yanlin Liu**: Data curation, **Shaozhi Fu:** Conceptualization, Writing-Reviewing & editing, Funding acquisition.

## Declaration of competing interest

The authors declare that they have no known competing financial interests or personal relationships that could have appeared to influence the work reported in this paper.

## Supplementary Material

Supplemental Material

## Data Availability

All data needed to support the conclusions are present in the paper and/or the Supplementary Materials. Additional data related to this paper may be requested from the authors.
