# Supplementary material for: Intraperitoneal administration of thermosensitive hydrogel Co-loaded with norcantharidin nanoparticles and oxaliplatin inhibits malignant ascites of hepatocellular carcinoma
Source: Drug Deliv. 2022 Aug 16;29(1):2713–22. doi: 10.1080/10717544.2022.2111480 (PMC9387330; doi:10.1080/10717544.2022.2111480)
Supplement: Supplemental Material [file IDRD_A_2111480_SM0001.docx]

**Supplementary Figures**

**
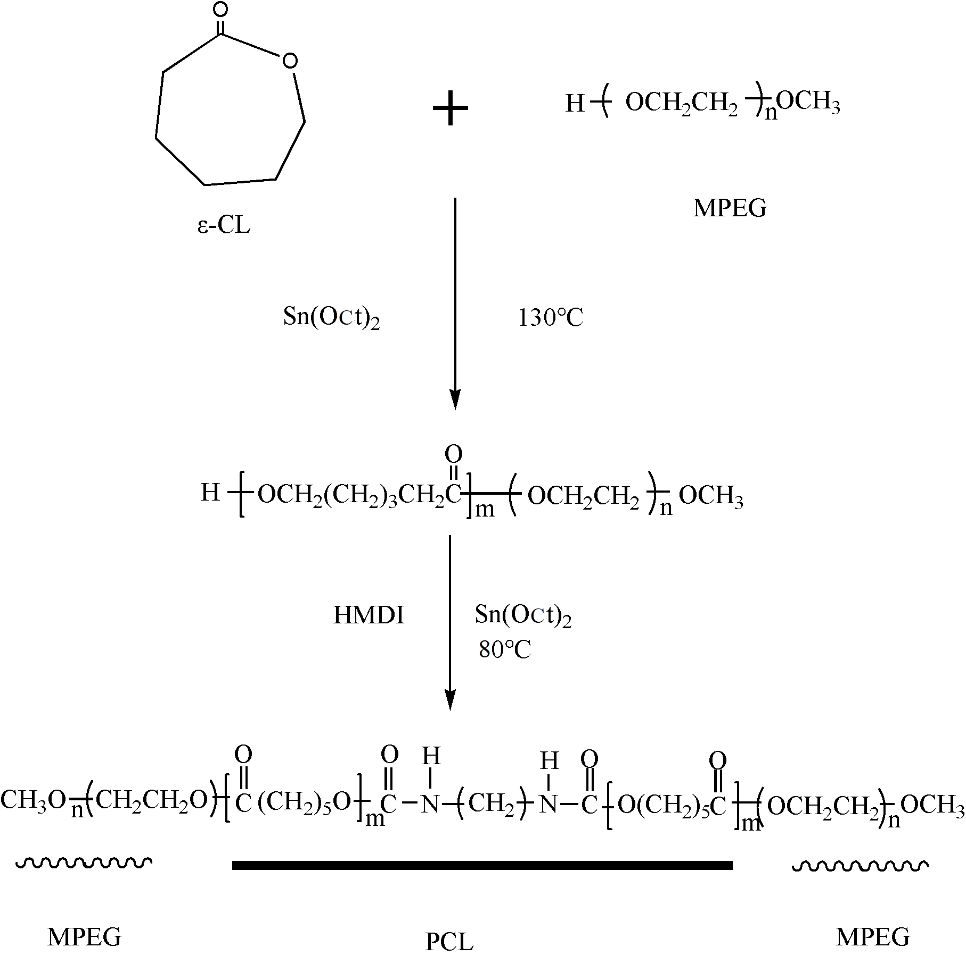
**

**Figure S1.** Chemical reaction routes of the PECE copolymer.

**
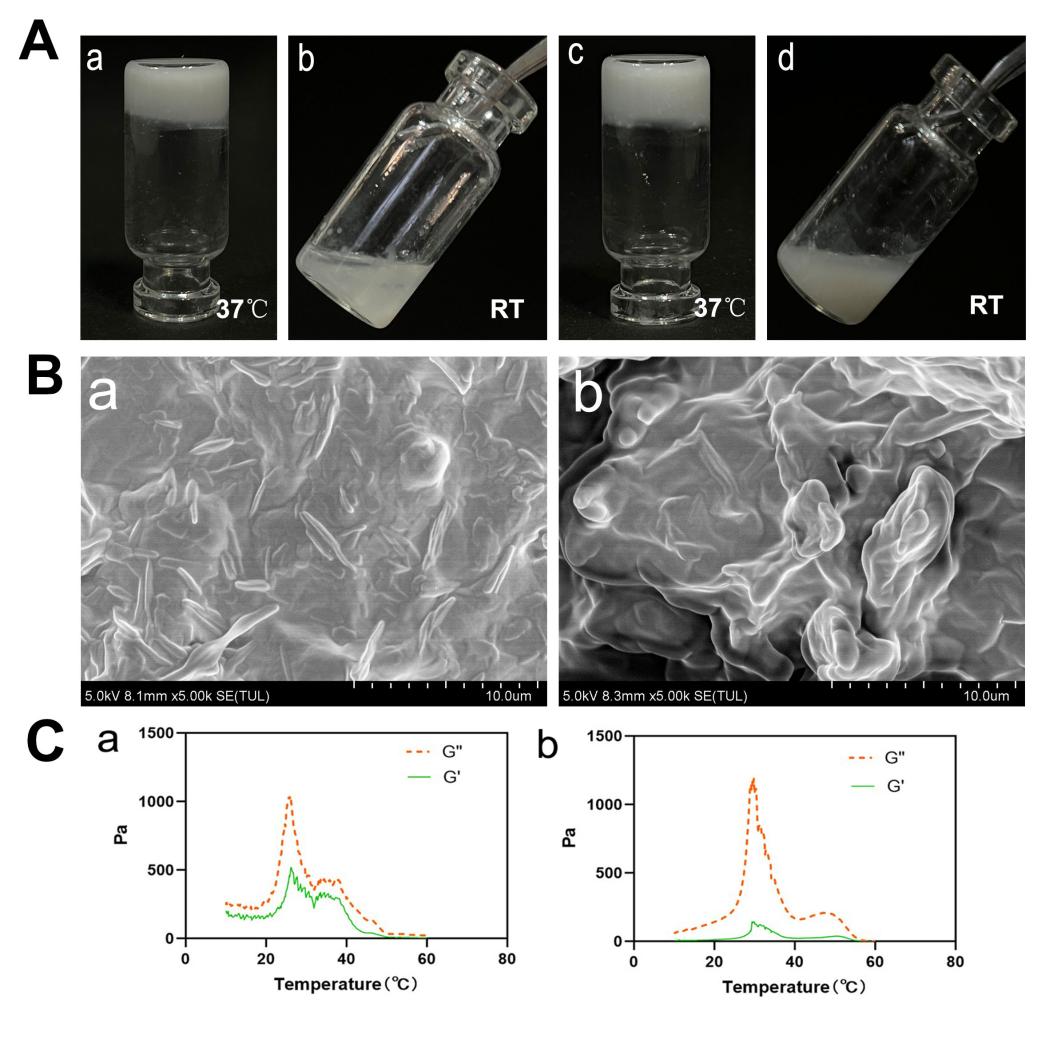
**

**Figure S2.** Characterization of blank PECE Hydrogel and drug-loaded hydrogel composite (N/O/Hydrogel). (A) The photos of the blank PECE hydrogel (a) and the N/O/Hydrogel (c) at 37 ^o^C; photos of the blank PECE hydrogel (b) and the N/O/Hydrogel (d) at room temperature (RT). (B) The SEM images of the freeze-dried PECE hydrogel (a) and N/O/Hydrogel (b). (C) The changes in storage modulus (G’) and loss modulus (G’’) of the blank PECE hydrogel (a) and the N/O/Hydrogel (b) by rheological analysis.
